# Current Role of CT Pulmonary Angiography in Pulmonary Embolism: A State-of-the-Art Review

**DOI:** 10.3390/jimaging10120323

**Published:** 2024-12-15

**Authors:** Ignacio Diaz-Lorenzo, Alberto Alonso-Burgos, Alfonsa Friera Reyes, Ruben Eduardo Pacios Blanco, Maria del Carmen de Benavides Bernaldo de Quiros, Guillermo Gallardo Madueño

**Affiliations:** 1 Radiology Department, University Hospital La Princesa, Calle Diego de Leon n. 62, 28006 Madrid, Spain; alfonsa.friera@salud.madrid.org (A.F.R.); rubeneduardo.pacios@salud.madrid.org (R.E.P.B.); mariadelcarmende.benavides@salud.madrid.org (M.d.C.d.B.B.d.Q.); 2 Radiology Department, Clinica Universidad de Navarra, Calle Santa Marta n. 1, 28027 Madrid, Spain; alonso@unav.es (A.A.-B.); ggallardom@unav.es (G.G.M.)

**Keywords:** computed tomography pulmonary angiography (CTPA), dual-energy CT (DECT), pulmonary embolism (PE), thrombotic burden, artificial intelligence (AI), risk stratification

## Abstract

The purpose of this study is to conduct a literature review on the current role of computed tomography pulmonary angiography (CTPA) in the diagnosis and prognosis of pulmonary embolism (PE). It addresses key topics such as the quantification of the thrombotic burden, its role as a predictor of mortality, new diagnostic techniques that are available, the possibility of analyzing the thrombus composition to differentiate its evolutionary stage, and the applicability of artificial intelligence (AI) in PE through CTPA. The only finding from CTPA that has been validated as a prognostic factor so far is the right ventricle/left ventricle (RV/LV) diameter ratio being >1, which is associated with a 2.5-fold higher risk of all-cause mortality or adverse events, and a 5-fold higher risk of PE-related mortality. The increasing use of techniques such as dual-energy computed tomography allows for the more accurate diagnosis of perfusion defects, which may go undetected in conventional computed tomography, identifying up to 92% of these defects compared to 78% being detected by CTPA. Additionally, it is essential to explore the latest advances in the application of AI to CTPA, which are currently expanding and have demonstrated a 23% improvement in the detection of subsegmental emboli compared to manual interpretation. With deep image analysis, up to a 95% accuracy has been achieved in predicting PE severity based on the thrombus volume and perfusion deficits. These advancements over the past 10 years significantly contribute to early intervention strategies and, therefore, to the improvement of morbidity and mortality outcomes for these patients.

## 1. Introduction

In the last few years, we have observed one of the most remarkable transformations in the field of medicine. The approach to pulmonary embolism (PE) has swiftly progressed in terms of both its diagnosis and treatment. The complexity of this condition necessitates a multidisciplinary approach, with the PERTs (Pulmonary Embolism Response Teams), developed in the mid-2010s, leading the way. Advances in diagnostic tools now allow for more precise risk stratification based on clinical, laboratory, and imaging parameters, turning each patient into a unique case that requires personalized decisions tailored to their individual scenario. The broad spectrum of nuances we encounter underscores the importance of collaboration across various specialties to optimize the therapeutic options for each patient’s specific condition [1].

In this regard, diagnostic radiology, more specifically computed tomography pulmonary angiography (CTPA), has evolved in parallel, providing new perspectives not only in achieving a more refined diagnostic approach but also by proving to be a valuable tool for patient risk stratification and therapeutic management, thus facilitating decision-making, as we will see in the development of this article. The technological advancements in CTPA have achieved an image quality that was unimaginable just over a decade ago. Coupled with the application of new artificial intelligence tools, this is a fitting moment to pause and reassess our current position regarding CTPA in relation to the complex landscape of PE and its present utility.

This narrative review seeks to provide an update on the new tools and techniques that are currently available, focusing on the data obtained from CTPA in emergency departments (ED). It addresses the challenge of data extraction from urgent and emergency studies, where speed and diagnostic accuracy often take precedence over in-depth volumetric analysis. The present work has been divided into sections based on the points we believe have evolved the most in recent years, or for which updating them can be of great value regarding the current diagnosis or treatment of PE. It is organized into five sections that include: (1) the quantification of thrombotic burden with CTPA; (2) its role as a predictor of mortality; (3) the new diagnostic techniques that are available; (4) the possibility of analyzing the thrombus composition to differentiate its evolutionary stage; and (5) the applicability of artificial intelligence in CTPA. The quantification of the thrombotic burden and its distribution can be useful, as both can be predictors of mortality for risk stratification in these patients and are useful for planning the most appropriate treatment. The applicability of CTPA, in both its single-energy and dual-energy modalities, has significantly evolved over the last 10 years, from occupying a purely diagnostic role to allowing the possibility of determining the composition of thrombi. Finally, the agility provided by the application of artificial intelligence to these aspects necessitates an update on what CTPA can contribute to the diagnosis, prognosis, and treatment of pulmonary thromboembolism.

Therefore, the aim of this review is to conduct a literature assessment of the advancements in the role of CT in both the diagnostic and prognostic aspects of pulmonary embolism.

## 2. CTPA Technical Approach

Typically, these studies are conducted using multidetector scanners without cardiac synchronization. Conventional computed tomography (CT) uses X-rays with a single energy level to generate images, providing anatomical detail but with limitations in differentiating tissues with similar compositions. In contrast, a dual-energy CT (DECT) uses two different energy levels, allowing for improved tissue characterization by distinguishing between tissues like calcium and fat or contrast agents. This enhances the diagnostic accuracy in specific clinical scenarios. In fact, although the use of DECT has been developing for over 10 years, its application in diagnosing pulmonary embolism has only recently become a valuable tool. It enhances the diagnostic performance of doctors with precise perfusion maps and allows for characterizing the thrombus’s evolutionary stage, adding therapeutic value. Table 1 and Table 2 summarize examples of CTPA protocols for PE studies using conventional CT and DECT in our institutions.

## 3. Methodology

After assessing the current literature and guidelines, the most rapidly evolving domains in the diagnosis of PE with CTPA were selected. Five topics were chosen for inclusion in this review: the quantification of the thrombotic burden, mortality predictors, new diagnostic techniques, the thrombus composition, and artificial intelligence, all within the context of diagnosis using CTPA. Each topic was reviewed by selecting guidelines, original articles, randomized controlled trials (RCTs), meta-analyses, and high-quality reviews published on PubMed/EMBASE, Web of Science, or Google Scholar from 2000 to 2024. Older but highly cited material was also considered.

## 4. Quantification of Thrombotic Burden with CTPA

The concept of maximizing the information gleaned from CTPA during the initial diagnostic evaluation of PE, often carried out in the emergency department, is not a novel one. Considering the urgent nature of this environment, the prompt interpretation and reporting of findings are of paramount importance. It is noteworthy that, currently, echocardiography is the only imaging method recommended by clinical guidelines for the accurately stratification of a patient’s risk during the course of a PE [2]. This tool offers more reproducible, reliable, and specific data concerning the right ventricle (RV) workload. Although factors such as vasoconstriction caused by the thrombus and the pre-existing functional state of the RV are crucial, bedside echocardiography provides detailed information about the RV’s condition at a particular moment. This reason is why it stands as one of the three fundamental pillars for stratifying patient risk.

The concept of quantifying thrombotic burden using CTPA is useful, as it aids in identifying and assessing the right ventricular (RV) overload, which is critical for the clinical management of patients. During the 2000s, three authors—Qanadli, Mastora, and Ghanima [3,4,5]—made significant contributions to this field, with varying degrees of clinical applicability. They focused on translating the thrombotic burden observed in CTPA studies into quantifiable and reproducible data. Terms such as “bilateral” or “massive” can often be ambiguous when applied to the thrombotic burden. Therefore, it is essential to have a clear and objective method for conveying this information to clinicians to ensure a consistent understanding. The term “bilateral” does not necessarily imply greater severity, and “massive” lacks a clear cutoff point, when applied to the thrombotic burden, that accurately defines the true impact on the pulmonary arterial bed. By providing more reproducible data, we can better assist in risk stratification and the development of tailored treatment plans.

Qanadli and Mastora both sought to establish correlations between the severity of pulmonary arterial obstruction and data obtained from echocardiography and angiography. Qanadli devised a formula that integrates the sum of the product of the proximal thrombus value (determined by the number of segmental arteries it originates from, with a minimum of 1 and a maximum of 20) and the degree of obstruction (ranging from 0 to 2). These data were compared with Miller’s arteriographic index [6], demonstrating over 90% correlation with control angiographies for patients with obstructions exceeding 40% and RV dilation on their echocardiography (RV/LR ratio > 0.6). Conversely, obstructions less than 40% are less likely to show ventricular dysfunction on echocardiography [3].

Mastora proposed a similar scoring system but provided greater granularity by categorizing artery lumen occlusions into five levels: less than 25%, 25–49%, 50–74%, 75–99%, and 100%. The cumulative data from the mediastinal, lobar, and segmental arteries allow for a determination of the central, peripheral, or global severity. Obstruction percentages less than 50% were found to correlate with mean pulmonary pressures of 20–60 mmHg, whereas those above 50% correlated with pressures between 30 and 80 mmHg [4].

Ghanima proposed a more straightforward scoring system that demonstrates better prognostic accuracy compared to previous models, thereby enhancing risk stratification [5]. This score is significantly associated with the Qanadli score and its accuracy is comparable in quantifying pulmonary arterial obstruction (*p* > 0.001) [7]. The score delineates four zones within the pulmonary arterial tree: subsegmental, segmental, lobar, and main. Although it does not consider the degree of obstruction, it suggests that more central embolizations correlate with greater severity, irrespective of the thrombus obstruction [4]. Ghanima identified significant relationships between proximal occlusion, the pulmonary artery obstruction index (measured using Qanadli’s technique), and the RV/LV ratio. Based on these findings, Ghanima advocates for his score as a prognostic marker for swift risk stratification in patients with PE.

However, Ghanima recently co-authored a compelling study from the TROLL registry [8,9], which confirmed a suspicion among experts in this field. It was found that peripheral thrombi are as lethal as central ones, despite the latter presenting a more dramatic diagnosis. Employing the developed scoring system, Ghanima observed inconsistent associations between high (proximal) and low (distal) scores and mortality rates. Surprisingly, patients with a score of 1 exhibited higher all-cause mortality at 30 days compared to those with a score of 4. Moreover, patients with a score of 4 received more systemic thrombolysis and were more frequently admitted to the intensive care unit (ICU), which potentially altered the natural progression of the disease. This study calls for a reevaluation of the current focus on central thrombectomies, which might overshadow patients who could benefit from catheter-directed fibrinolysis. Caution is advised, as finding a physiological rationale for these results is complex, and there might be underlying confounding factors. The authors emphasize the significance of semi-quantitative measurement of the thrombus burden, volume quantification, and iodine mapping to assess pulmonary perfusion, with the goal of predicting long-term sequelae post-PE, such as pulmonary hypertension.

## 5. CTPA as a Predictor of Mortality

The established predictors of mortality in PE include the initial clinical severity, hemodynamic parameters, biomarkers such as proBNP and troponin I, and evidence of RV dysfunction on echocardiography. Additionally, factors such as the patient’s age, comorbidities, and other risk conditions (including chronic obstructive pulmonary disease, cardiovascular disease, and cancer) are considered, as outlined in the PESI and sPESI (simplified Pulmonary Embolism Severity Index) scoring systems. However, findings from CTPA are not currently included among the mortality predictors and are therefore not part of contemporary risk stratification models. Acute RV dysfunction occurs in approximately 34% of cases at disease onset and is considered one of the key indicators for intermediate-risk PE [2,10,11].

In the past decade, substantial scientific efforts have been made to integrate CTPA data into risk assessments, recognizing that many findings from the initial CTPA used to diagnose PE are often underutilized. Most studies have focused on evaluating RV metrics as prognostic factors [12,13,14,15,16,17,18]. For example, Kang et al. [12] conducted a significant study involving 260 patients with acute PE, identifying RV dysfunction indicators such as the position of the interventricular septum, contrast reflux into the inferior vena cava, and the RV/LV diameter ratio in the axial and four-chamber views, as well as the 3D RV/LV volume ratio (RVV/LVV ratio). They concluded that the 3D ventricular volume is a predictor of early mortality in these patients, regardless of any clinical risk factors or comorbidities. The other parameters also predicted adverse outcomes, except for the axial RV/LV diameter ratio being greater than 1, which did not. The study highlighted that 3D volumetric measurements were superior to other RV dysfunction signs observed through CTPA in predicting adverse outcomes and 30-day mortality. Patients with an RVV/LVV ratio greater than 1.2 experienced adverse events or death at 30 days six times more frequently than those with a ratio below 1.2, of whom 97% survived [12].

**Figure 1:**
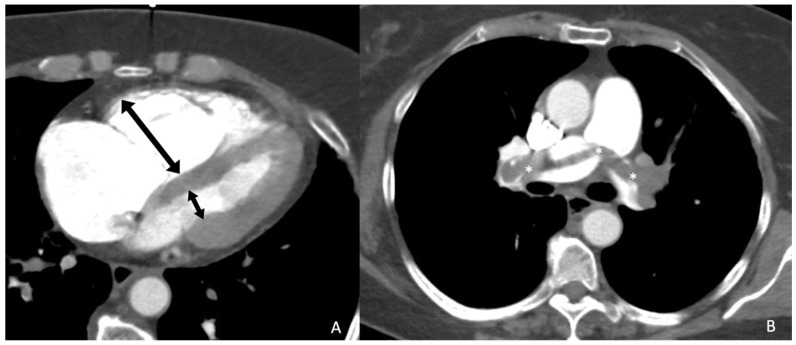
Fifty-six-year-old woman diagnosed with acute pulmonary thromboembolism, by axial CT angiography. (**A**). Axial RV/LV diameter ratio > 1 measured at the base of both ventricles (black arrows). (**B**). Filling defects in both main pulmonary arteries (*), with a saddle thrombus.

Conversely, the meta-analysis by Meinel et al. [13], published in 2015, reviewed 49 studies involving 13,162 patients and concluded that the axial RV/LV ratio should be included in all reports (Figure 1). An axial RV/LV ratio greater than 1 is associated with a 2.5-fold higher risk of all-cause mortality and adverse outcomes, and a 5-fold higher risk of PE-related mortality. This finding contrasts with the results of Kang et al., as we have previously mentioned, since this author asserts that this datum is not a predictor of adverse effects. However, Meinel et al. provide a detailed analysis of the relationship between the axial RV/LV diameter ratio and the all-cause mortality risk, stratified by sources of heterogeneity, resulting in a robust conclusion. In clinical practice, especially in the urgent context where CTPA studies are conducted, the ratio of the right ventricular diameter to that of the left is a measure that is easy to obtain, unlike ventricular volumes or four-chamber reconstructions. Therefore, Meinel’s recommendation is deemed appropriate. While the parameters suggested by Kang et al. are undeniably valuable, their acquisition post-acute event requires practice, time, and skill, which may not be feasible in emergency settings.

Lastly, it is important to mention the PE-SCORE developed by Weekes et al. [19], which is designed as a prognostic tool to predict clinical deterioration or death in the days following diagnosis. This score incorporates nine clinical, laboratory, and imaging variables related to right ventricular function, including the RV/LV diameter ratio on CT (Table 3). A PE-SCORE above 6 predicts a high probability of clinical deterioration or death with greater reliability than sPESI at 5 days post-diagnosis, according to the same author [20]. Despite demonstrating excellent short-term results, it is highly likely that similar studies will emerge in the coming years, including those with external validation, that further support the use of this promising tool in managing this condition. Such studies may guide decisions on early outpatient care and the need for more aggressive interventions when necessary.

## 6. New Diagnostic Tools with CTPA

Recent publications have increasingly highlighted the use of innovative techniques that facilitate a more objective quantification of pulmonary perfusion. Zhao et al. [21] offer a comprehensive account of the validation of an automated method designed to quantify the hypoperfused pulmonary territory due to pulmonary embolism (PE) in pigs. This method employs the minimum-cost path (MCP) technique and shows promise for application in risk stratification. The study successfully validated the use of the MCP technique to quantify the tissue territory distal to PE that leads to total occlusions, using the resultant ischemic territory as a reference point. These findings demonstrate that the MCP technique can accurately and automatically quantify the distal territory of PE associated with pulmonary arterial obstruction by only utilizing CTPA image data. Consequently, this technique holds the potential to offer a more precise assessment of PE severity by quantifying the total mass of at-risk tissue caused by PE.

In recent years, significant advancements have been made in DECT, particularly with the development of single-source DECT featuring rapid tube voltage switching and dual-source DECT. These innovations facilitate high-quality, near-simultaneous imaging at two distinct energy levels (typically 80 and 140 kV). By utilizing the differing attenuation properties of iodinated contrast in these scans, it becomes possible to quantify the iodine concentration, thereby allowing assessment of the perfusion status of the pulmonary parenchyma. Moreover, DECT’s capability to perform spectral and material decomposition imaging further enhances its diagnostic and prognostic accuracy in the evaluation of pulmonary embolism (PE). The term ‘spectral’ refers to the system’s ability to acquire images at multiple wavelengths or energy levels, enabling the collection of more detailed information about the body’s tissues and structures, as different materials and pathological conditions may respond differently to diverse wavelengths. DECT, also known as multispectral CT, offers several advantages in the diagnosis and assessment of PE:

1. Perfusion mapping: DECT generates lung perfusion maps, which are valuable in identifying perfusion defects corresponding to embolized areas. This functional information complements anatomical details, enhancing the diagnostic accuracy;
2. Iodine quantification: the ability to measure the distribution of iodine allows for the assessment of blood flow and perfusion deficits, providing additional insights into the extent of vascular occlusion and potential ischemia;
3. Characterization of the thrombus evolutionary stage: DECT can help differentiate between acute and chronic thrombi by analyzing their compositions. Acute thrombi exhibit higher iodine contents, while chronic ones show decreased attenuation.
4. Reduction of the radiation dose: DECT protocols can potentially reduce the amount of radiation exposure by eliminating the need for multiple phases of contrast-enhanced scans, such as pre- and post-contrast imaging.

**Figure 2:**
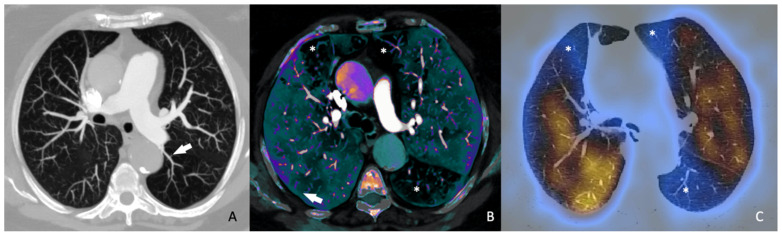
Eighty-nine-year-old woman diagnosed with chronic pulmonary thromboembolism. (**A**) Axial CT angiography (maximum intensity projection—MIP—reconstruction) showing severe narrowing in the superior segmental artery of the left lower lobe (white arrow) as sequela of PE. (**B**) Fusion image of CT angiography and color-coded iodine density showing wedge-shaped perfusion defects (*) in the middle lobe, lingula, and left lower lobe, with the latter corresponding to the findings in image (**A**). (**C**) SPECT-CT fusion image showing wedge-shaped perfusion defects (*) similar to those obtained with dual-energy CT (**B**).

Further studies also underscore the importance of DECT [22,23,24,25,26]. As previously mentioned, these systems acquire data at two energy levels, enabling the differentiation of tissues with varying attenuation properties, which is especially beneficial for substances like calcium and iodine that exhibit high attenuation. The differentiated materials are visualized through their decomposition, and each material’s concentration can be calculated using an absorption algorithm. Different materials demonstrate distinct behaviors at various energy levels, allowing for more effective differentiation compared to single-energy spectrum imaging. This capability enables the creation of “iodine maps” (Figure 2), which specifically illustrate the presence of iodine in different slices. DECT can thus enhance the detection of pulmonary embolisms and aid in stratifying their severity [25]. On these maps, the distribution of iodine is directly proportional to the blood volume, allowing for the creation of pulmonary blood volume (PBV) maps. Defects distal to emboli are not commonly detected, as they occur more frequently (82–95%) in occlusive emboli compared to non-occlusive emboli (6–9%). Consequently, these defects are considered indicators of severity. Numerous studies have shown that a higher number and a larger size of defects in the PBV correlate with adverse outcomes, such as increased pulmonary arterial obstruction indices and RV dysfunction, which is assessed based on whether the patient has an RV/LV diameter ratio greater than 1 [25]. However, Im et al. [24] found that only the RV/LV ratio was a higher risk factor for all-cause mortality at 30 days, and not quantitative PBV measurement, in their propensity score matching comparing DECT with conventional CT. More recently, Lee et al. [23] published findings on the quantitative analysis of pulmonary perfusion, comparing the relative PBV values (%PBVs) and normalized PBVs (PBVms) among patients based on their pulmonary density, and discovered a significant correlation between the PBVm and sPESI.

## 7. Thrombus Composition and Resolution

Understanding the composition of a thrombus is crucial in tailoring therapeutic approaches to the treatment of it. The composition of a thrombus is closely linked to its age and, consequently, its structural organization. Older thrombi typically exhibit a denser fibrin network and may contain calcified elements or cholesterol crystals derived from atherosclerotic plaques. This increased density can diminish the effectiveness of both pharmacological interventions, such as thrombolysis, and mechanical procedures, like thrombectomy. Additionally, older clots are more likely to incorporate a neutrophil extracellular trap, which further complicates treatment by enhancing the clot’s resistance to breakdown. These considerations underscore the importance of early intervention and the potential necessity for more aggressive or combination therapies in managing older, more complex thrombi [27]. This allows clinicians to adjust their treatment strategies and minimize the risk of associated adverse events. Red blood cell (RBC)-dominant clots tend to respond better to intravenous thrombolysis with tissue plasminogen activator (tPA), leading to faster clot dissolution (typically younger thrombi). Conversely, fibrin/platelet (F/P)-dominant clots are more resistant to this treatment, often requiring a mechanical thrombectomy with advanced techniques, such as stent retrievers or aspiration devices (usually older thrombi) [27,28]. Furthermore, the thrombus composition influences the likelihood of fragmentation during mechanical extraction, which could lead to distal embolization and worsened outcomes. Fibrin-rich thrombi are generally tougher, increasing the risk of complications during their retrieval, whereas RBC-rich thrombi are more fragile and easier to remove in one pass [27].

Leonhardi et al. [29] have introduced the concept of “thrombus texture analysis” as a potential prognostic marker in PE. Drawing on the characterization of cerebral thrombi in patients suffering from ischemic stroke, a direct correlation analysis between histological and imaging characteristics in both non-contrast and contrast-enhanced CT scans has been undertaken. This imaging technique has the potential to predict the thrombus permeability, which could be particularly beneficial for mechanical thrombectomies in ischemic stroke, as the composition of the thrombus has shown statistically significant correlations with treatment outcomes [30,31]. The composition and age of the thrombus may have implications for reperfusion outcomes in both fibrinolytic and mechanical aspiration treatments. In their research, Leonhardi et al. aimed to determine if there is an association between thrombus texture characteristics in PE and clinical parameters and mortality, utilizing software that analyses CTPA images obtained during the diagnosis of patients with PE. This study is preliminary, although it presents promising future possibilities, and further prospective studies with larger sample sizes are required to draw more definitive conclusions.

The role of DECT in the characterization of thrombi has been extensively documented in the literature, particularly in studies concerning acute ischemic stroke. Jiang et al. investigated the utility of DECT in analyzing the thrombus compositions in patients with acute ischemic stroke (AIS) [32]. They examined thrombi from 88 patients with AIS, categorizing them as either RBC-dominant or F/P-dominant. The key findings show that RBC-dominant thrombi exhibited higher virtual non-contrast (VNC) values and lower iodine concentrations (ICs), slopes of the spectral Hounsfield unit curve (λHUs), and effective atomic numbers (Zeffs) compared to F/P-dominant thrombi. The CT density on IC images proved to be the most accurate parameter for distinguishing between thrombus types, with an area under the curve (AUC) of 0.94, showing 77.78% sensitivity and 100% specificity. These results suggest that DECT-derived parameters, especially the CT density on IC images, could serve as biomarkers for thrombus composition, aiding in personalized thrombectomy strategies [32].

Another significant point to consider, often overlooked, is that a high percentage of thrombi are physiologically lysed. However, although it is challenging to specify the exact rate of this, the average incidence of chronic pulmonary hypertension secondary to PE is estimated to be around 3.4% (95% CI 2.1–4.4%) [33]. It is also pertinent to highlight a series of studies that demonstrate the degree of thrombus resolution over time [33,34,35]. Ak et al. [34] present a noteworthy prospective study published in 2022, involving 290 patients, where the overall estimated probability of complete resolution was 42% at 7 days, 56% at 10 days, and 71% at 45 days. This resolution occurred more rapidly in patients with peripheral thrombi and in patients with cancer, although the latter group experienced a significantly higher mortality rate. There is no standardized follow-up protocol for patients with PE, so there are no follow-up data to substantiate these results, and thrombus lysis is influenced by numerous factors, meaning the resolution time can vary from patient to patient. In fact, although several studies have explored this topic, Ak et al. are the first to do so in a prospective manner. For instance, Aghayeb et al. [35] report a resolution rate of 68.8% in patients at 3 months, increasing to 94.1% beyond this period. Other authors, such as Van Es et al. [36] and Van Rosum et al. [37], report lower resolution rates, with 44% at 21 days and 32% at 42 days, respectively.

## 8. Artificial Intelligence in PE

The rapid evolution of artificial intelligence (AI) and machine learning (ML) has brought about a transformative shift in the analysis of medical imaging, including CTPA. The potential for the use of AI in CTPA extends beyond diagnosis; it can also enhance prognostication. By combining AI-derived image analysis with clinical risk scores (e.g., PESI, sPESI), we can refine our ability to identify high-risk patients who may benefit from more aggressive treatments, such as thrombolysis or mechanical thrombectomy. AI algorithms can assist radiologists in:

1. Automated detection: AI algorithms can automatically detect PE on CTPA scans with high accuracy, reducing the burden on radiologists and ensuring prompt diagnosis;
2. Quantification of the thrombus burden: AI can provide precise measurements of the thrombus burden and its distribution throughout the pulmonary vasculature, enhancing risk stratification. Automated quantification can reduce interobserver variability and facilitate a more standardized approach to interpreting CTPA results;
3. Improved workflow: AI can streamline the workflow in radiology departments by prioritizing studies based on their clinical urgency and flagging critical findings for immediate review by radiologists. This can lead to quicker diagnoses and treatment decisions, especially in emergency settings;
4. Integration with clinical data: machine learning models can integrate CTPA findings with patient demographics, clinical history, and biomarkers to create predictive models that assess the risk of adverse outcomes in patients with PE.

In recent years, numerous researchers have been actively developing techniques to automate the assessment of thrombus loads, aiming to correlate these with traditional methods through the application of AI [38,39,40,41]. The objective is to validate these novel methods. One significant contribution is by Xi et al. [38], who have introduced a scoring system designed to quantify the thrombus load using CTPA and a deep learning (DL) algorithm that is specifically for PE risk stratification. This method, published in 2024, employs scores as described by Qanadli [3] and Mastora [4], in addition to a thrombus ratio (defined as the ratio between the thrombus volume and the volume of the pulmonary artery on CTPA) and thrombus volume. This is based on a deep learning convolutional neural network (DL-CNN) algorithm developed by Liu et al. [39]. The retrospective study included all patients diagnosed over the course of a year at their institution (*n* = 70), with a 30-day follow-up, classified according to the 2019 European Society of Cardiology (ESC) criteria [2]. The study is thorough, incorporating the patients’ clinical and analytical variables such as sPESI, creatine kinase-MB, troponin T, and N-Terminal pro B-type natriuretic peptide levels, as well as their PaO2/FiO2 ratios. Additionally, CTPA examines thrombus load data alongside other cardiovascular parameters like the ventricular diameters and areas in axial views, and the diameters of the pulmonary and aortic arteries. The findings reveal that their DL-CNN model demonstrated superior accuracy in predicting high and intermediate-high risk PE patients, particularly for hemodynamically stable individuals. There was a significant correlation between the thrombus ratio, the PaO_2_/FiO_2_ ratio, and RV load, highlighting the potential of this model as a predictor for acute RV failure.

Their results included a correlation analysis between the thrombus ratio and other parameters, as well as between the thrombus load and risk stratification. The thrombus ratio showed the highest efficacy in identifying high and intermediate-high risk patients. This was followed by the thrombus volume and the scores from the method of Qanadli and Mastora (with AUC values of 0.719, 0.695, 0.688, and 0.652, respectively). Although no statistically significant differences were noted among these four measures, the thrombus ratio showed the most consistent performance in predicting high-risk patients and aiding in risk stratification. It was the only marker that demonstrated a statistically significant difference in hemodynamically stable patients, proving valuable for identifying those at risk of clinical deterioration. The study underscores a link between the thrombus load, RV dysfunction, and risk stratification. An advantage of this thrombus ratio score is its automation, real-time processing, time efficiency, and reduced observer dependency compared to the scoring method by Qanadli and Mastora [38].

Lanza et al. have also contributed significantly to this field, describing their findings using a nnU-Net algorithm for the detection of PE, particularly central PE, and to measure patients’ blood clot volume (BCV) in automated severity stratification [41]. They utilized the RSPECT dataset from the Radiological Society of North America (RSNA) Pulmonary Embolism CT dataset, training an algorithm on 205 PE cases and 340 negative cases. The test set included 6573 exams, with 1888 positives for PE. Their data revealed significant differences in the BCV between negative and positive cases. Statistical analysis indicated a strong correlation between the BCV and the presence of PE, central PE, and an increased right to left ventricle ratio (RV/LV), with a *p*-value of less than 0.0001. These results suggest that the BCV is a significant indicator for detecting PE and central PE, as well as for assessing right ventricular overload. The accuracy and predictive values of their model suggest its potential to enhance the diagnosis and clinical management of PE. Implementing such an algorithm can significantly improve the efficiency and accuracy of PE diagnosis, thereby reducing the workload of radiologists and potentially leading to quicker treatment decisions and better patient outcomes. This underscores the increasing importance of integrating AI into clinical practice to improve healthcare delivery.

## 9. Conclusions

This review emphasizes the evolving role of CTPA in the diagnosis and prognosis of PE, highlighting its value in quantifying the thrombotic burden and predicting mortality risk. The RV/LV ratio has been validated as a significant prognostic factor associated with an increased mortality risk. Advanced techniques such as DECT have substantially enhanced diagnostic accuracy, detecting perfusion defects that may be missed by conventional CTPA. Additionally, AI is transforming CTPA interpretation, improving the detection of smaller emboli and optimizing the assessment of PE severity. However, challenges remain, including the standardization of AI algorithms across diverse clinical settings and their effective integration into routine practice. Further research should focus on developing new AI models tailored to specific PE subtypes and patient demographics, as well as conducting large-scale multicenter studies to validate these models in varied clinical environments. Incorporating additional clinical data, such as genetic markers or biomarkers related to thrombosis, may further refine risk stratification and enhance the predictive accuracy for PE. Addressing these issues will be essential for advancing PE management and improving patient outcomes in the clinical setting.

## Figures and Tables

**Table 1:** Examples of protocols for planning a CTPA for the study of pulmonary thromboembolism using single-energy and dual-energy, and radiation dose parameters used in authors’ hospitals.

	General Electric (Revolution EVO)	Siemens (Somaton Drive)
Scan mode	Single energy (128)	Dual energy (2 × 128)
Scan area	Diaphragm to lung apex	Diaphragm to lung apex
Scan direction	Caudo-cranial	Caudo-cranial
Scan time (s)	3.32 s	9 s
Tube voltage (kVp)	100	100/140 (A/B) (tin filter)
Tube current (ref. mAs)	130	71/60 (A/B)
Dose modulation CARE Dose 4D	-	CARE Dose 4D
CTDI vol (mGy)	8.5	6
Rotation time (s)	0.4	0.33
Pitch	0.98	0.55
Slice collimation (mm)	0.625	0.6
Acquisition (mm)	128 × 0.4	128 × 0.6

CTDI (computed tomography dose index); s (seconds); kVp (kilovoltage peak); mAs (milliampere-seconds); mGy (miliGray); A/B (refers to each different tube of the dual-energy CT scan).

**Table 2:** Example of protocol for the administration of iodinated contrast in both single-energy and dual-energy used in authors’ hospitals.

Iodine concentration	300 mg/mL
Contrast media volume (mL/kg)	1.5
Contrast media flow rate (mL/s)	4
Bolus timing	Bolus tracking
Bolus tracking threshold (HU)	100
ROI position	Pulmonary trunk
Scan delay (s)	6
Saline flush volume (mL)	40
Saline injection rate (mL/s)	4
Needle size (G)	18
Injection site	Antecubital vein

HU (Hounsfield units); ROI (region of interest); G (gauge).

**Table 3:** PE-SCORE. Primary outcome probability for final model variables [19].

Variable	Adjusted Odds Ratio	Development Database	Validation Database	Points Assigned
		Relative Risk	Relative Risk	
Creatinine > 2.0 mg/dL	5.37	2.48	2.16	2
Dysrhythmia	4.00	2.39	3.67	1
Suspected/confirmed systemic infection	3.47	2.63	3.67	1
Systolic blood pressure < 100 mmHg	2.87	2.65	2.85	1
Abnormal heart rate (<50 or >100 beats/min)	2.26	2.17	1.67	1
Syncope	1.97	2.00	2.25	1
Medical or social reason for hospitalization	1.91	2.00	1.76	1
Echocardiography with abnormal RV	1.81	2.67	3.16	1
CT RV:LV ratio elevated	1.73	2.23	2.38	1
Total Points PE-SCORE score (minimum = 0; maximum = 10 points)				

Abbreviations: CT = computed tomography; LV = left ventricle; RV = right ventricle.

## Data Availability

The original contributions presented in this study are included in the article. Further inquiries can be directed to the corresponding author(s).
